# Possible Immunosuppressive Effects of Drug Exposure and Environmental and Nutritional Effects on Infection and Vaccination

**DOI:** 10.1155/2015/349176

**Published:** 2015-04-05

**Authors:** H. P. Huemer

**Affiliations:** Medical University of Innsbruck, Department of Hygiene, Microbiology & Social Medicine, Fritz-Pregl-Straße 3, 6020 Innsbruck, Austria

## Abstract

A variety of drugs which are not primarily considered to be immunosuppressive agents have been described to modulate the humoral and cellular immune response in humans or animals. Thereby they may have an influence on the effectiveness and possible side effects of vaccines. This mini review lists some of the different substance classes and also some of endogeneous, infectious, nutritional, and environmental influences with suspected capability to interfere with immunizations. Studies in most cases focused on substances with known immunosuppressive functions, but there is growing evidence for immunomodulatory effects also of commonly used drugs with wide distribution. In particular combinations of those antiproliferative and antiphlogistic side effects of different substance classes have not been studied in detail but may substantially interfere with the development of a functional humoral and cellular immune response. The drugs of importance include antipyretics, anticoagulants, tranquilizers, and substances influencing lipid metabolism but also commonly used drugs of abuse like alcohol or cannabinoids. Additional substances of environmental, nutritional, or microbiological origin may also play a role but their combinatory/synergistic effects have been disregarded so far due to the lack of systematic data and the complex study designs necessary to elucidate those complex epidemiologic questions.

## 1. Introduction

Besides classical immunosuppressive drugs, which are not topic of this minireview, the number of lower grade agents with, often neglected, immunosuppressive activity is surprisingly high. As they include many different classes and various principles and mechanisms of action only some examples are listed here.

## 2. Antiphlogistic Substances

*Nonsteroidal anti-inflammatory drugs* (NSAIDs) are widely used as a first line minor pain medication and also for their antipyretic effects in acute febrile infections. In addition to their anti-inflammatory function they often may have also complex immunological effects on cell proliferation, migration, antibody, and cytokine production.

Thus aspirin, acetaminophen, and ibuprofen have been shown to interfere with antiviral immune functions influencing the duration of viral shedding in rhinovirus infected humans [1].

The mechanism of action seems to affect also very basic cellular antiviral functions as in the mouse model both aspirin and paracetamol decreased the interferon induced antiviral responses against influenza virus of cultured mammalian cells [2].

The specific immune response is also affected as a variety of NSAIDs have been shown to inhibit the antibody production in human cells [3]. Such mechanisms appear to be predominantly mediated by the inhibition of cyclooxygenase-2 (COX-2) and, for example, lead to a reduction of the antibody response against the smallpox vaccine (vaccinia virus) response also in the mouse model [4].

Due to the wide use of NSAIDs against rheumatic diseases these findings could definitely contribute to the fact that the vaccination efficiency in elderly humans often is quite moderate [5].

But those mechanisms may be also important in younger ages as an earlier trial showed a clear reduction of the antibody response to vaccinations in children after paracetamol treatment, independent of the documented febrile responses [6].

Aspirin not only interferes with the development of an antibody response [3] but also leads to immune tolerance in dendritic cells and numerous other immunomodulatory effects in different immune cells (for review see [7]). Also the development of a cellular immune response seems to be impaired by aspirin or ibuprofen as can be concluded by reduction of the skin reactivity against mycobacterium PPD in rats [8].

It is evident that aspirin also leads to a significant increase in mortality in experimental animal studies, for example, with influenza virus, but surprisingly randomised studies in humans on a larger scale do not exist [9]. This is surprising, as high dose aspirin treatment, as suggested by US physicians, has been speculated to have contributed to the high mortality rates of the flu pandemic in 1918 [10, 11].

## 3. Antianxiety Drugs and Psychopharmacology

*Benzodiazepines* are also widely used substances. They are used as sedative, anxiolytic, anticonvulsive, and muscle relaxing drugs. They act as modulators of the gamma-amino-butyric-acid (GABA) receptors in the central nervous system (CNS) which are the most important inhibitory receptors in the CNS [12]. GABA receptors consist of several subunits, not all of them interacting with benzodiazepines. Sedative effects of benzodiazepines are mediated by *α*1-subunits and anxiolytic and anticonvulsant effects by the *α*2- and *α*3-subunits [13]. The classical benzodiazepines like diazepam (ValiumR) show all of the typical effects; more recently introduced substances are more selective GABA receptor agonists in order to reduce side effects such as memory impairment and abuse potential [14].

In addition to the receptors in the CNS, there exist also peripheral benzodiazepine receptors. Also known as 18 kDa translocator protein (TSPO) they are multimeric complexes of 18 kDa proteins found in most peripheral organs in high density. They are localized in the outer mitochondrial and have been associated with increased steroid synthesis and transport of cholesterol to the inner mitochondrial membrane and with increases in cell proliferation such as cancer and gliosis, but also tissue repair and immunomodulating functions [15]. In the CNS, they are mainly expressed by activated microglial cells and are considered to play a role also in neuroinflammatory processes [16]. TSPO ligands have been shown in experimental models to have anti-inflammatory effects [17, 18].

The data about benzodiazepines leading to immune suppression are not many. Old reports postulated that peripheral benzodiazepine receptors may be involved in the development of immune cells in neonatal rats [19] and diazepam and methamphetamine have been found to suppress the antibody production and T-cell function in mice [20]. In animal models we found that diazepam had a profound negative effect on the resistance of mice against poxvirus infection as well as the development of a virus specific antibody response. Additionally the proliferative response of human lymphocytes/spleen cells was also inhibited by benzodiazepines [21]. Evidence for a direct immunomodulatory action for benzodiazepines emerged also from studies that demonstrated the presence of TSPO on immune/inflammatory cells and diazepam effects were discussed as being related to the number of TSPO sites present on immune cells [22].

## 4. Anticoagulants

### 4.1. Sulfated Oligosaccharides

Sulfated oligosaccharides are involved in numerous biological interactions including cellular adherence, oligomerization of cell growth factors, protein localisation at cell surfaces, the control of proteolysis, angiogenesis, and tumor metastasis. Heparin like other negatively charged polysulfates (e.g., dextran sulfate) in addition to their anticoagulatory effects therefore has numerous effects on the specific and unspecific immune system (for review see [23, 24]). This includes interference with cellular functions and cytokine production as well as direct binding to factors of the humoral immune defense like, for example, chemokines or complement factors [25, 26].

Surprisingly these functions are that potent that heparin can be used under certain conditions as an anti-inflammatory therapy even in severe medical conditions like, for example, inflammatory bowel disease [27]. Also low molecular heparinoids appear to have significant immunosuppressive and anti-inflammatory effects in animal studies making them likely targets for drug development [28].

### 4.2. Salicylic Acid

Concerning the long term use of low dose* aspirin* as anticoagulant there are no extended studies in humans evaluating possible immunosuppressive effects.

## 5. Lipid Metabolism

Lipid metabolism plays an important role in immune dysfunction.

### 5.1. Bioactive Lipid Mediators

Bioactive lipid mediators like immunosuppressive prostaglandins have been shown to play an important role in critical illnesses and inflammation (for review see [29]). It has been also well established that uptake of certain unsaturated lipids has a modulating effect on inflammatory responses [30, 31]. Therefore it is not too surprising that substances regulating lipid metabolism may have an influence on immune mechanisms, as has been shown for the statins in numerous studies.

### 5.2. Statins

Statins interfere with the expression of proinflammatory cytokines as well as CRP; interference with the MHC presentation of antigens and a decrease in the inflammatory response have been observed in several studies, including modulation of neutrophil function or cytokine expression, but the exact mechanisms still have to be elucidated [32].

Despite observed anti-inflammatory effects it appears that statins can both stimulate and suppress the immune system. These effects have been shown to mitigate autoimmune diseases by downregulating Th1 helper cells but on the other hand might influence allergic reactions presumably by increasing IgE levels [33].

Inhibitory effects on the Th1 immune response but also presumable stimulatory effects on Th2 responses could have implications on vaccination strategies as has been suggested recently [34].

However, data of a stimulation of the antibody response was not observed in a subsequent publication [35]. Thus more extended studies in humans would be needed.

The suppressive effects of statins are now well established and their use has been associated with lower mortality from influenza [36] and other immune driven mortality associated with community acquired pneumonia [32]. Therefore statins and other immunomodulatory drugs have been suggested recently to be beneficial and recommended for use under those conditions [37].

## 6. Drugs of Abuse

There is a long history of records that drug abuse is clearly associated with increased levels of infections (for review see [38]).

### 6.1. Alkohol

Alcohol is one of the widest distributed drugs with known immunosuppressive functions [39]. A lot of clinical and experimental data provide evidence that exposure to ethanol exhibits a variety of effects on immune cell functions. Ethanol decreases lymphocyte responsiveness to mitogens, suppressing chemotactic and phagocytic functions, and alters the production of cytokines by lymphoid cells. One important mechanism of ethanol action is upon macrophage function which is also the case with tobacco or environmental pollutants [40].

### 6.2. Cannabinoids

The endocannabinoid system has a variety of complex functions including the response to environmental stimuli and emotional functions. Working mainly via the cannabinoid (CB1) brain receptors endocannabinoids can regulate the release of neurotransmitters such as GABA and glutamate, thereby influencing numerous mechanisms leading to addiction. In addition to those central cannabinoid receptors also a peripheral cannabinoid receptor (CB2) has been subsequently detected. CB2 is expressed predominantly in immune cells and cannabinoids therefore have received increasing interest as potentially immunomodulatory drugs recently [41]. Thus the endocannabinoid system can regulate immune responses and both plant-derived and synthetic cannabinoids have been shown to signal through CB1 or CB2 via G proteins [42, 43].

Effects of high concentrated cannabis ingestion on serum immunoglobulin and complement protein concentrations as well as decrease in functional subsets of peripheral blood lymphocytes and NK cells have been reported in high school students [44]. The immunosuppressive effects are not only described for the main psychoactive component of cannabis, that is, tetrahydrocannabinol (THC) but also described for less psychoactive cannabinoids like cannabidiol (CBD) as inhibitory effects of CBD on the functional activity of splenocytes and antigen driven antibody production have been shown in the mouse model [45]. The immunosuppressive effects can be quite severe also in healthy young adults, influencing the course of cowpox virus infection or side effects of the vaccinia (smallpox vaccine) virus [46].

### 6.3. Other Drugs of Abuse

Immunosuppressive effects have also been reported for* amphetamines* or* cocaine* and among other mechanisms seem to rely predominantly on cellular cytotoxicity and inhibition of phagocyte functions [47, 48].

## 7. Microbiological Antigens and Nutritional Effects

### 7.1. Bacterial Mechanisms

It is known that bacteria, protozoa, and also helminths exert a wide array of immunosuppressive mechanisms. This is not only due to pathogenic organisms but also due to commensals which have to find a way to survive hostile environments.

The mechanisms of actions are very diverse including a variety of toxins and bacterial cell wall components like* lipopolysaccharides* [49] or lipids [50] mostly acting via toll-like receptors (TLR).

Also CpG-rich bacterial DNA motives alone have been shown to lead to a reduction of the adaptive T-Cell immune response via TLR-9 and activation of the immunosuppressive pathway of tryptophan catabolism [51].

The fact that vaccination success is reduced in patients infected with mycobacteria is known for long time [52]. One of the possible mechanisms has been suggested by the finding that the systemic suppression of IFN-gamma response resolves after excision of the infected areas [53].

### 7.2. Fungal Toxins

Also fungal* mycotoxins* of various genera of fungi have been shown to have immunosuppressive functions mostly affecting cellular immune responses [54]. Thus aflatoxins most recently have been shown in animal experiments to downregulate cytokines production by splenic lymphocytes [55] and a reduced antibody response to vaccines has been repeatedly observed following mycotoxin food contamination in broiler chicken [56].

A possible role of nutritional uptake of probiotics in immunosuppression or stimulation is still under dispute as many of them exert anti-inflammatory effects on the immune system including induction of regulatory T-cells [57]. In addition to findings that probiotics can attenuate allergic reactions or inflammatory bowel disease there is consensus that the* gut microbiome* in general may have a big influence on the outcome of immune responses and vaccination [58].

### 7.3. Polyphenols

Phytochemicals like curcumin, ginkgo, and others are present in many fruits or herbs and commonly used as spices and they have also been shown to have profound immunomodulatory and antiphlogistic effects [59]. These include regulation of cellular transcription factors and inhibition of nitrite oxidase as well as inhibition of release of cytokines like IL1beta or TNF alfa [31, 60].

### 7.4. Anti-oxidants

Besides flavonoids numerous other substances, many of those among the* food antioxidants* [61], have been included in the list of potentially anti-inflammatory and immunosuppressing food additives and suggested for therapeutic use as disease modifying “nutricals” (for review see [62]).

## 8. Environmental Chemicals

### 8.1. Pesticides

Chemicals like* pesticides* in food stuffs or the frequently found* arsenic* in drinking water have been clearly shown to have detrimental effects on the immune system leading to increased rates of infection and cancer [63, 64].

Additionally there are numerous new chemical compounds in our daily life which have been suspected to have negative influences on the human immune system and therefore might exert synergistic effects with other low grade “immunosuppressants.”

### 8.2. Perfluorinated Compounds (PFCs)

Perfluorinated compounds (PFCs) have been recently found associated with a reduced humoral immune response upon childhood vaccination [65] but combinatory effects with other substances have not been studied so far. PFCs are fluorocarbon derivatives but unlike the PCBs banned in the 80s they are more resistant to biodegradation and are therefore very widely distributed environmental pollutants.

### 8.3. Bisphenol A

Bisphenol A, a substance present in polycarbonates used also in the food industry, has been shown in animal experiments to reduce the cytokine response and immunoglobulin titers after vaccination [66].

### 8.4. Heavy Metals

Heavy metals like cadmium or lead in drinking water reduced the antibody production in mice [67] and also have an influence on the spleen and thymus cell repertoire as well as the acquired immune response [68].

## 9. Combinatory/Synergistic Effects

Only very few studies/observations have reported synergistic effects of low level suppressants and commonly used drugs due to the complex study designs that would be necessary for epidemiologic investigations and the lack of information in routine medicine.

### 9.1. Benzodiazepines and Cox-Inhibitors

This is a very common combination in human medicine. A recent publication has shown that the combination of midazolam and a cyclooxygenase-2 inhibitor is able to inhibit the lipopolysaccharide-induced interleukin-6 production in human peripheral blood mononuclear cells even if each drug separately did not show an effect [69].

### 9.2. Benzodiazepines and Cannabis

In our previous findings we experienced a young patient with an unusually severe cowpox infection but did not show a proper seroconversion and development of specific antibodies. This was attributed to the mixed drug taking habits of the otherwise healthy young male [70]. Subsequent animal experiments testing the function of benzodiazepines and whole cannabis as well as fractions revealed a clear effect of both substances on the cellular and humoral immune functions which can also act in synergy [21, 46].

### 9.3. Sulfated Oligosaccharides and NSAID

The combination of heparin and aspirin has been found suitable for the treatment of recurrent spontaneous abortion (RSA) associated with immune alterations [71]. Similarly low molecular weight heparin plus low dose aspirin resulted in a higher birth rate than intravenous immunoglobulins in the treatment of antiphospholipid antibody syndrome in women with recurrent abortions [72].

## 10. Summary

A variety of drugs which are not primarily considered to be immunosuppressive have been described to modulate the humoral and cellular immune response in humans or animals. Thereby they may have an influence on the effectiveness and possible side effects of vaccines.

This mini review tried to give some examples of a growing variety of different substance classes with suspected or proven capability to interfere with immunizations.

While there are numerous studies investigating the influence of established immunosuppressive therapies on vaccinations, including chemotherapeutics as well as biologicals like TNF antagonists (for review see [73]), the influence of an increasing variety of widely used drugs especially of drug combinations remains unclear. Therefore highly alerted physicians and epidemiologists are necessary to elucidate those often neglected “side effects” which might become also the main effects under certain circumstances [74]. In particular cumulative effects of combinations of different suppressive principles have to be considered as they may reach an immunosuppressive “threshold” which could not be expected with single medications.
